# Glycosidases Heterogeneity Among Dimethylhydrazine Induced Rat Colonic Tumours

**DOI:** 10.1038/bjc.1974.186

**Published:** 1974-09

**Authors:** N. Mian, D. M. Cowen, C. A. Nutman

## Abstract

**Images:**


*Br. J. Cancer* (1974) **30**, 231

### GLYCOSIDASES HETEROGENEITY AMONG DIMETHYLHYDRAZINE INDUCED RAT COLONIC TUMOURS

N. MIAN, D. M. COWEN AND C. A. NUTMAN

*From the Department of Experimental Pathology and Cancer Research, School of Medicine, Leeds LS2 9NL, England*

Received 10 April 1974. Accepted 13 May 1974

**Summary.—Activities of N-acetyl-β-D-glucosaminidase, N-acetyl-β-D-galactosaminidase, β-D-galactosidase and α-L-fucosidase were measured in rat colonic tumours induced by 1,2-dimethylhydrazine. Tumours varied considerably in their enzyme content, not only from different animals but also from the same animals. Enzymatic heterogeneity among tumours appeared to be related to their site of origin in the colon. The descending colon, which after the DMH treatment showed a significant increase in the levels of glycosidases, also gave rise to a larger number of adenocarcinomata than other parts of the colon. The relative changes in the activities of four glycosidases seemed to show a good correlation.**

IN GENERAL, rat colonic tumours induced by 1,2-dimethylhydrazine (DMH) indicated a significant increase in the levels of some glycosidases compared with the remaining colonic mucosa of the treated animals (Mian and Cowen, 1974). Evidence of a generalized change, as indicated by an overall reduction of $^{35}S$ uptake, in parts of the DMH treated rat colon where tumours were commonly developed has been given (Springer, Springer and Oehlert, 1970). The site where tumours would arise in the mouse colon after DMH treatment could be predicted with great certainty (Haase *et al.*, 1973). These authors observed that in the experimental mice the last 4 cm of the colon was always the site of at least one polyp. A gradient in the cell turnover times from the proximal to the distal end of the colon and a considerable variation in the cell proliferation rates in the DMH induced mouse colonic tumours and in the primary adenocarcinomata of the human large bowel have already been observed (Sawicki and Rowinski, 1970; Haase *et al.*, 1973; Bottomley and Cooper, 1973; Camplejohn, Bone and Aherne, 1973).

The present work was carried out to assess the enzymatic heterogeneity among the DMH induced rat colonic tumours with reference to the site specificity and variations in tumour morphology and cell kinetics.

### MATERIALS AND METHODS

*Animals.*—Wistar male rats, about 12 weeks old, were obtained from Bantin and Kingman, Hull, England. The animals were fed Oxoid 41B (Oxo Ltd, London, England) and water *ad libitum*. The experimental animals were injected subcutaneously for 26–32 weeks with a weekly dose of 20 mg/kg body weight of 1,2-dimethylhydrazine dihydrochloride (DMH) in 4% solution of $Na_2$. EDTA made freshly each time as described by Haase *et al.* (1973). The control animals were given $Na_2$. EDTA in saline solution. Injection of animals was stopped one week before they were used for the experiment.

*Preparation of tissue homogenates.*—Animals were starved overnight before the experiment but had unrestricted access to water. They were killed by cervical dislocation. Colons were removed, opened by a longitudinal slit and washed with ice cold saline. Total length of the colon, location of the tumours and their number were recorded. Tumours were excised and kept individually. The remaining colons of the tumour bearing animals and the control colons were cut into 8 equal segments. They are designated as follows: first 4 segments starting from the anal margin as the descending colon, 5th segment as the transverse colon and the last 3 as the ascending colon. The mucosa was scraped off with a microscope slide. Tumours and mucosal scrapings were homogenized in ice cold saline containing 0·1% Triton X-100, giving 40 strokes with a Teflon pestle in Potter-Elvehjem homogenizer. Protein concentration of the homogenates was between 4 and 5 mg/ml.

Estimation of glycosidases—β-D-Galactosidase (EC 3.2.1.23) and α-L-fucosidase (EC 3.2.1.51), N-acetyl-β-D-glucosaminidase (EC 3.2.1.30) and of N-acetyl-β-D-galactosaminidase (EC 3.2.1.53)—were determined as previously described (Mian and Cowen, 1974).

The substrates used were p-nitrophenyl-β-D-galactoside, p-nitrophenyl-α-L-fucoside, p-nitrophenyl-N-acetyl-β-D-glucosaminide and p-nitrophenyl-N-acetyl-β-D-galactosaminide; p-Nitrophenol was used as a standard. Enzyme activities are expressed as nmoles of p-nitrophenol liberated/h/mg of protein. Total protein was determined using the biuret reaction method (Hubscher, West and Brindley, 1965). Crystalline bovine serum albumin was used as a standard.

*Chemicals.*—All chemicals used were A.R. grade. p-Nitrophenol, substrates, and crystalline bovine serum albumin were purchased from Sigma Chemical Co. Ltd, London. DMH (1,2-dimethylhydrazine dihydrochloride) was obtained from Aldrich Chemical Co. Inc., Wisconsin, U.S.A.

### RESULTS

Out of the 125 macroscopic colonic tumours used in the present work, 25 tumours were examined histologically. The examination of these specimens indicated that all these tumours were well differentiated adenocarcinomata, the majority being of the polypoid tubular type. Most of these tumours gave a negative reaction for PAS and alcian blue staining, indicating a lack of mucosubstances but occasionally extracellular pools of these substances were observed in some tumours.

Estimation of glycosidases of colonic tumours showed a large variation among the activities of individual tumours (Table I). The difference in the enzyme profile existed not only among tumours from different experimental animals but also from the same animals. Tumours arising in the descending colon showed a significant increase ($P < 0{\cdot}005$) in the enzyme levels compared with the enzyme contents of the adjacent colonic mucosa (Fig. 1–4). However, the distribution of the glycosidases in the colon of the control rats was found to be fairly uniform (values falling within the shaded areas in Fig. 1–4). Analysis of the data on the enzyme activities in the tumour bearing animals (Table II) indicated that 2 N-acetylhexosaminidases and β-D-galactosidase were elevated significantly in the descending and the transverse colon compared with the control values. α-L-Fucosidase showed a significant increase in the transverse colon and in certain regions of the descending colon. In the ascending colon of the experimental animals none of these glycosidases showed any significant rise compared with the controls.

The pattern of enzyme increase among tumours arising in different regions of the colon appeared to be correlated with the frequency of tumour incidence in those areas of the colon. The data from 55 experimental rats indicated that the frequency of the tumour incidence and the presence of multiple adenocarcinomata were considerably higher in the descending and the transverse colon than in the ascending colon (Fig. 5). Parts of the colon with a high frequency of tumour incidence also showed a significant increase

TABLE I.—*Glycosidases in Colonic Tumours Induced by 1,2-dimethylhydrazine. The Enzyme Activity was Expressed as nmoles of p-nitrophenol Released/h/mg Protein; 100 Tumours from 20 DMH Treated Rats were Studied*

| Enzyme | Mean ± s.e. | Range |
|---|---|---|
| N-acetyl-β-D-glucosaminidase | 21·22 ± 1·57 | 4·42–63·15 |
| N-acetyl-β-D-galactosaminidase | 13·34 ± 0·99 | 4·06–49·34 |
| β-D-galactosidase | 16·66 ± 1·15 | 5·02–57·23 |
| α-L-fucosidase | 4·84 ± 0·44 | 0·46–19·73 |

TABLE II.—*Levels of Significance of Enzyme Increase in the Colonic Segments of DMH Treated Animals Compared with the Respective Segments of the Control Rats were Calculated using Student's " t " Test. N.S. Means that Values were not Significant at* $P < 0{\cdot}005$ *Levels. The Actual Enzyme Activities are Plotted as Histograms in Fig.* 1–4

| Colonic segments | N-acetyl-β-D glucosaminidase | N-acetyl-β-D galactosaminidase | β-D-galactosidase | α-L-fucosidase |
|---|---|---|---|---|
| 1st | $P<0{\cdot}001$ | $P<0{\cdot}001$ | $P<0{\cdot}001$ | N.S. |
| 2nd | $P<0{\cdot}001$ | $P<0{\cdot}001$ | $P<0{\cdot}001$ | $P<0{\cdot}005$ |
| 3rd | $P<0{\cdot}001$ | $P<0{\cdot}001$ | $P<0{\cdot}001$ | N.S. |
| 4th | $P<0{\cdot}001$ | $P<0{\cdot}001$ | $P<0{\cdot}001$ | $P<0{\cdot}005$ |
| 5th | $P<0{\cdot}005$ | $P<0{\cdot}005$ | $P<0{\cdot}005$ | $P<0{\cdot}005$ |
| 6th | N.S. | N.S. | N.S. | N.S. |
| 7th | N.S. | N.S. | N.S. | N.S. |
| 8th | N.S. | N.S. | N.S. | N.S. |

FIG. 1.—Levels of N-acetyl-β-D-glucosaminidase in tumours and in colons of experimental and of control rats.

Abscissa: segments of colon (1st–8th) starting from the anal margin; ordinate: nmoles of p-nitrophenol released/h/mg protein. Open circles (○) show activities of the tumours plotted approximately in the same position on the abscissa where tumours were found *in situ*. Open columns (□) and shaded columns (▧) represent activities of enzyme of the experimental and of the control rats respectively. Each enzyme activity is mean ± s.e. 100 colonic tumours, 20 experimental and 16 control rats were studied.

in the levels of 2 N-acetylhexosaminidases and β-D-galactosidases compared with the ascending colon, where the comparative enzymatic changes were insignificant and the frequency of tumour incidence was also very low.

Although both tumours and the mucosa from different regions of the colons of the

FIG. 2.—Levels of N-acetyl-$\beta$-D-galactosiminidase in tumours and in colons of experimental and of control rats. Rest of description is the same as Fig. 1.

FIG. 3.—Levels of $\beta$-D-galactosidase in tumours and in colons of experimental and of control rats. Rest of description is the same as in Fig. 1.

FIG. 4.—Levels of α-L-fucosidase in tumours and in colons of experimental and of control rats. Rest of description is the same as in Fig. 1.

FIG. 5.—Frequency of tumour incidence in different parts of the colon.

Abscissa: segments of colon (1st–8th) starting from the anal margin; ordinate frequency of tumour incidence. Closed circles (●) show the presence of adenocarcinomata and open circles (○) the presence of multiple adenocarcinomata in the given segments. The frequency ratios were calculated from the data obtained from 55 rats which received from 20 to 32 weekly s.c. injections of 1,2-dimethylhydrazine. Total number of macroscopic tumours observed was 403 and the mean length of the colon was 22·3 cm.

experimental animals showed a large variation in their glycosidases content, the relative changes in the levels of these enzymes seemed to hold a good correlation. The correlation coefficient values ($r$) were near +1 (ranging from 0·8786 to 0·9790).

### DISCUSSION

The present work suggests that the DMH induced colonic tumours in rats varied a great deal in their glycosidase activity profile. N-acetyl-β-D-glucosaminidase, N-acetyl-β-D-galactosaminidase and β-D-galactosidase have been found to show a linear increase in their activities during the cell cycle in a synchronous population of L 5178 Y cells (Bosmann and Bernacki, 1970). A difference in the cell kinetics and/or chromosomal abnormalities of tumours which may alter the enzyme synthesis in the cell could explain the heterogeneity among tumours. Nevertheless, comparison of enzyme activity data of tumours arising in different parts of the rat colon also suggested that the enzymatic variation could be due to the site specificity. The apparently normal looking mucosa of the descending colon of the experimental animals which showed a highly significant increase ($P < 0·001$) in 2 N-acetylhexosaminidases and in β-D-galactosidase gave rise to tumours whose enzyme levels were further elevated significantly ($P < 0·005$) compared with the adjacent mucosa. In contrast to this, in the mucosa of the transverse colon, although these enzymes showed a significant increase ($P < 0·005$), the tumours produced here were either low

FIG. 6.—Colon from a tumour bearing rat given 24 weekly injections of DMH showing hyperplastic crypts lacking in muco-substances adjacent to the normal crypts. Haematoxylin PAS. × 258.

or similar in activities to the surrounding colonic mucosa.

As the frequency of tumour incidence in the descending and transverse colon was higher than in the ascending colon, a significant change in the glycosidases could be due to a high degree of malignant transformation of these regions. The presence of microscopic tumours and of hyperplastic crypts could be observed histologically in all areas of the colon (Fig. 6). Similar hyperplastic lesions in the colonic mucosa of the tumour bearing mice have been noted previously (Thurnherr *et al.*, 1973).

Although it is difficult to rule out whether the changes in the activities of the glycosidases were due to the chronic toxic effects of DMH, the differential change in various parts of the colon and a wide heterogeneity among tumours suggest that the observed biochemical alteration is primarily associated with neoplastic transformation. The final outcome is probably the result of the interplay of several factors, both intrinsic in the tumour organization and possibly secondarily to the luminal environment which changes progressively from the caecum to the anus.

A good correlation between the relative changes in the levels of different glycosidases in tumours and in the colonic mucosa of the experimental animals could also suggest that either transformation of the cell or the toxicity of the carcinogen hit some loci of the genome which regulate the synthesis of the enzymes involved in the hydrolysis of macromolecules containing glycosidic linkages. Further studies on the kinetics and the inhibition behaviour of these enzymes in rat colonic tumours and mucosa are under investigation.

We wish to thank Professor E. H. Cooper for his interest in this work and Elizabeth A. Batte for her skilled technical assistance. The work has been supported by the Yorkshire Cancer Research Campaign.

### REFERENCES

BOSMANN, H. B. & BERNACKI, R. J. (1970) Glycosidase Activity: Glycosidase Activity in L 5178 Y Mouse Leukemia Cells and the Activity of Acid Phosphatase, β-galactosidase and β-N-acetylgalactosaminidase and β-N-acetylglucosaminidase in a Synchronous L 5178 Y Cell Population. *Expl Cell. Res.*, **61**, 379.

BOTTOMLEY, J. O. & COOPER, E. H. (1973) Cell Proliferation in Colonic Mucosa and Carcinoma of the Colon. *Proc. R. Soc. Med.*, **66**, 1183.

CAMPLEJOHN, R. S., BONE, G. & AHERNE, W. (1973) Cell Proliferation in Rectal Carcinoma and Rectal Mucosa. A Stathmokinetic Study. *Eur. J. Cancer*, **9**, 577.

HAASE, P., COWEN, D. M., KNOWLES, J. C. & COOPER, E. H. (1973) Evaluation of Dimethylhydrazine Induced Tumours in Mice as a Model System for Colorectal Cancer. *Br. J. Cancer*, **28**, 530.

HUBSCHER, G., WEST, G. R. & BRINDLEY, D. N. (1965) Studies on the Fractionation of Mucosal Homogenates from the Small Intestine. *Biochem. J.*, **97**, 629.

MIAN, N. & COWEN, D. M. (1974) Glycosidases in Normal and Dimethylhydrazine Treated Rats and Mice with Special Reference to the Colonic Tumours. *Br. J. Cancer*, **29**, 438.

SAWICKI, W. & ROWINSKI, J. (1970) Proliferation Kinetics in Epithelium of Guinea-pig Colon: 1. Variations depending on Crypt Length and its Localization. *Cell Tiss. Kinet.*, **3**, 375.

SPRINGER, P., SPRINGER, J. & OEHLERT, W. (1970) Die Vorstufen des 1,2-Dimethylhydrazine-Unduzierten Dick- und Dunndarmcarcinoms der Ratte. *Z. Krebsforsch*, **74**, 236.

THURNHERR, N., DESCHNER, E. E., STONEHILL, E. H. & LIPKIN, M. (1973) Induction of Adenocarcinomas of the Colon in Mice by Weekly Injections of 1,2-Dimethylhydrazine. *Cancer Res.*, **33**, 940.


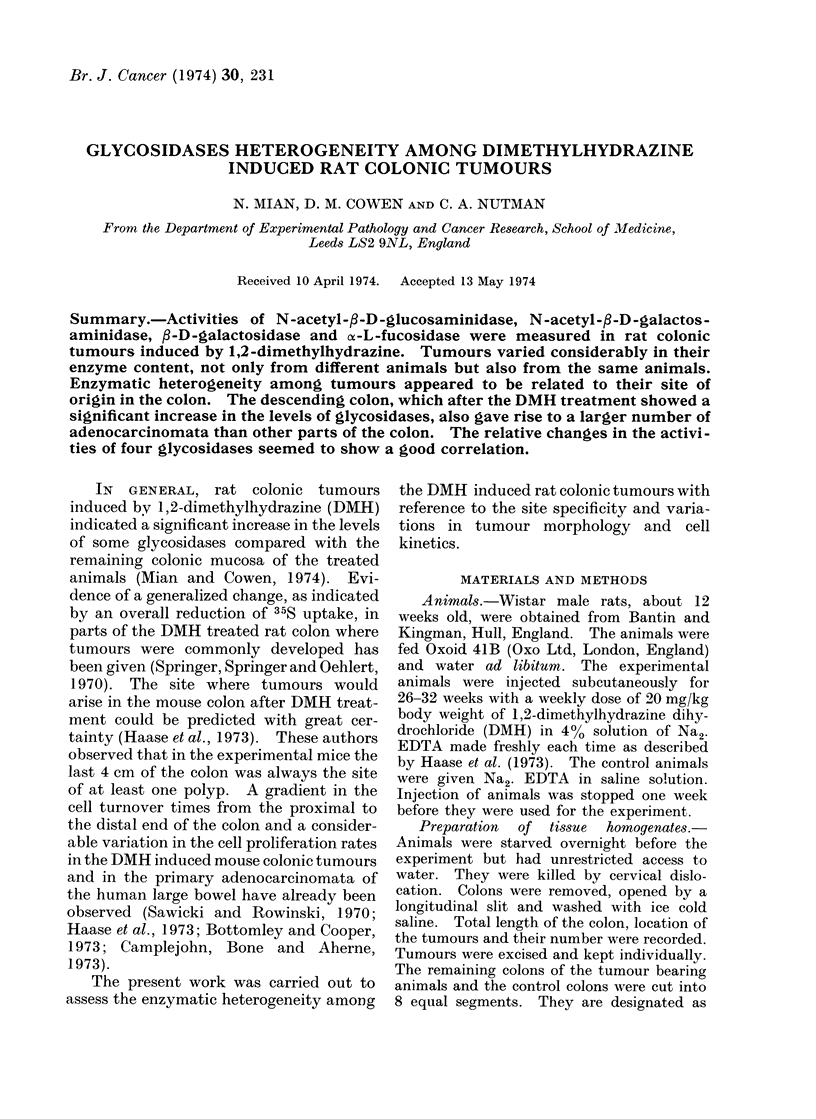

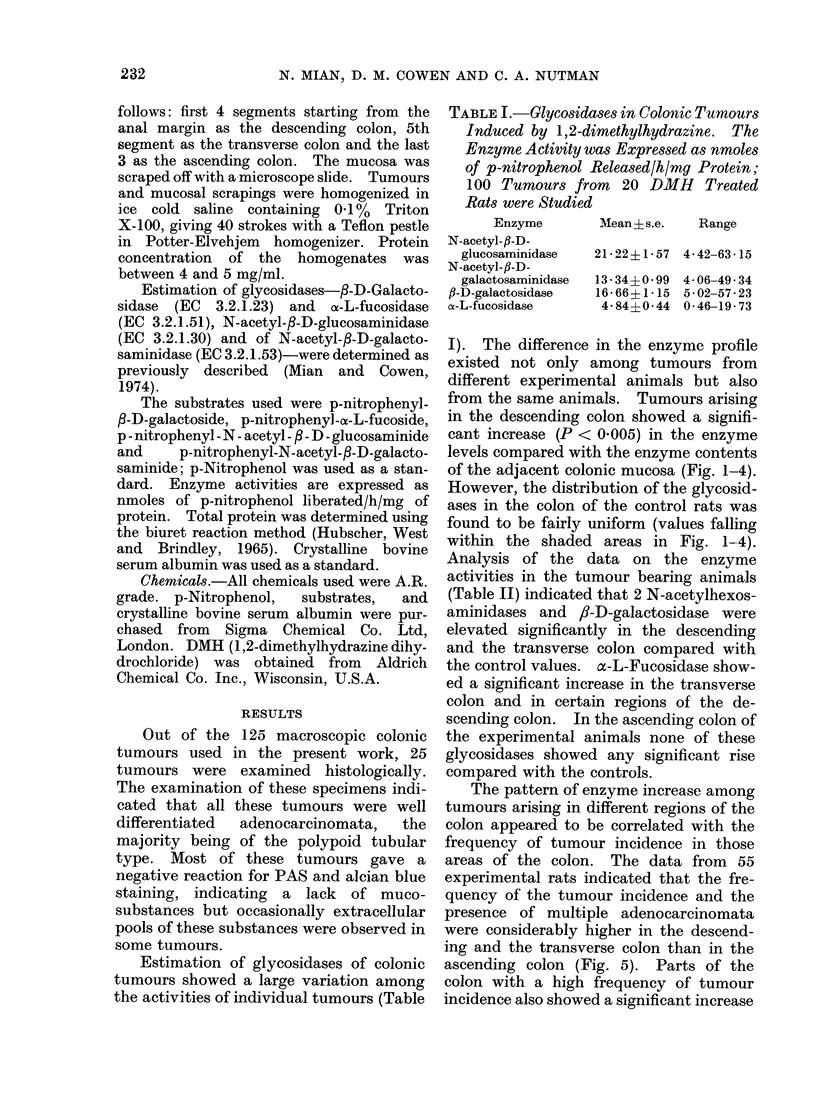

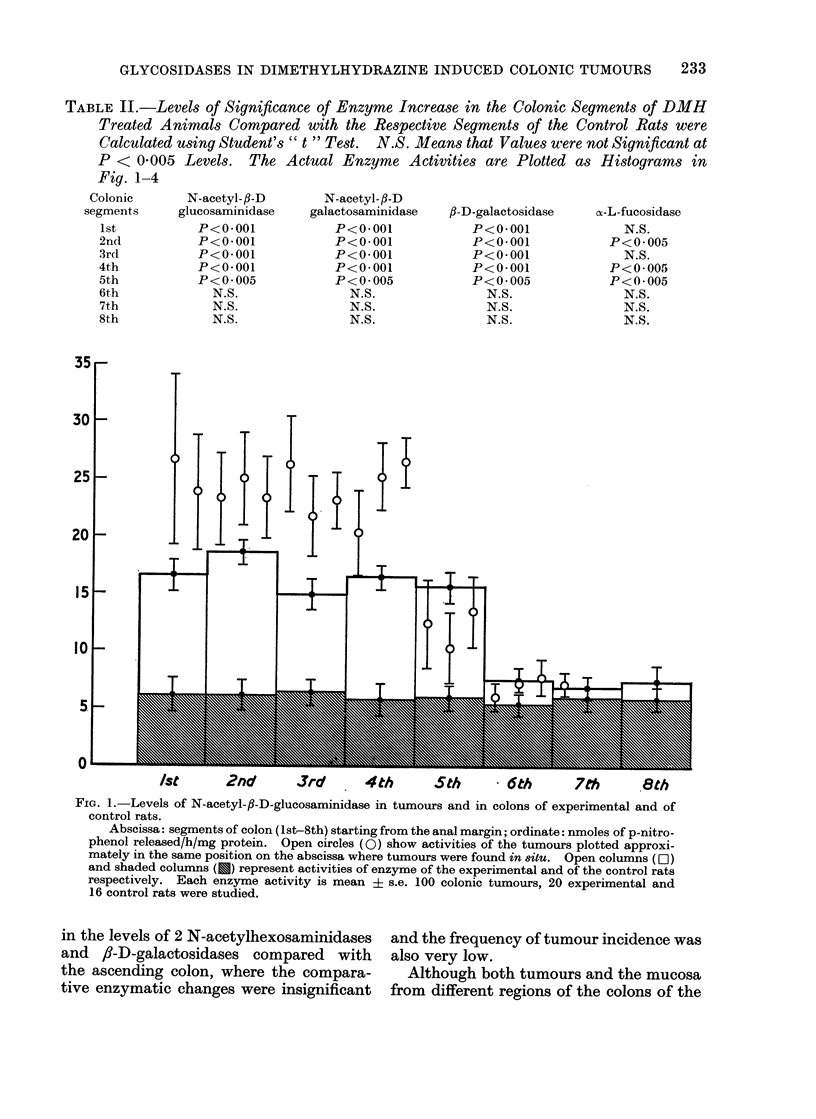

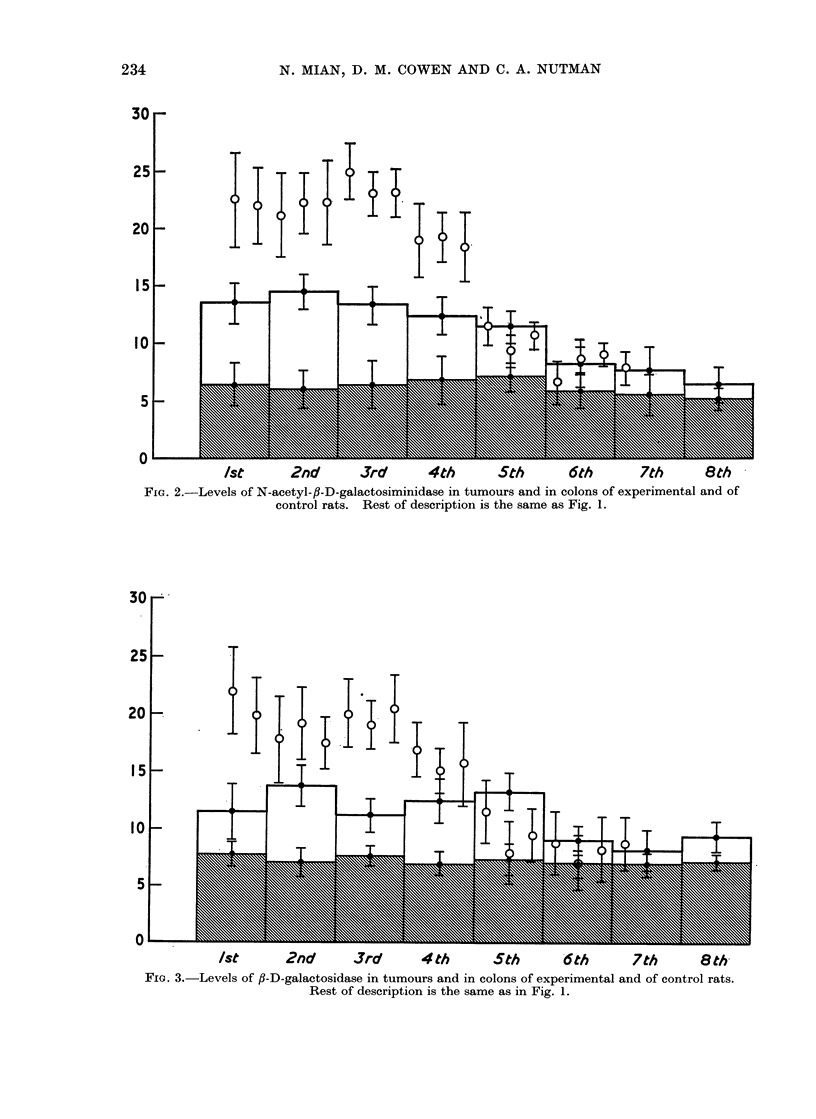

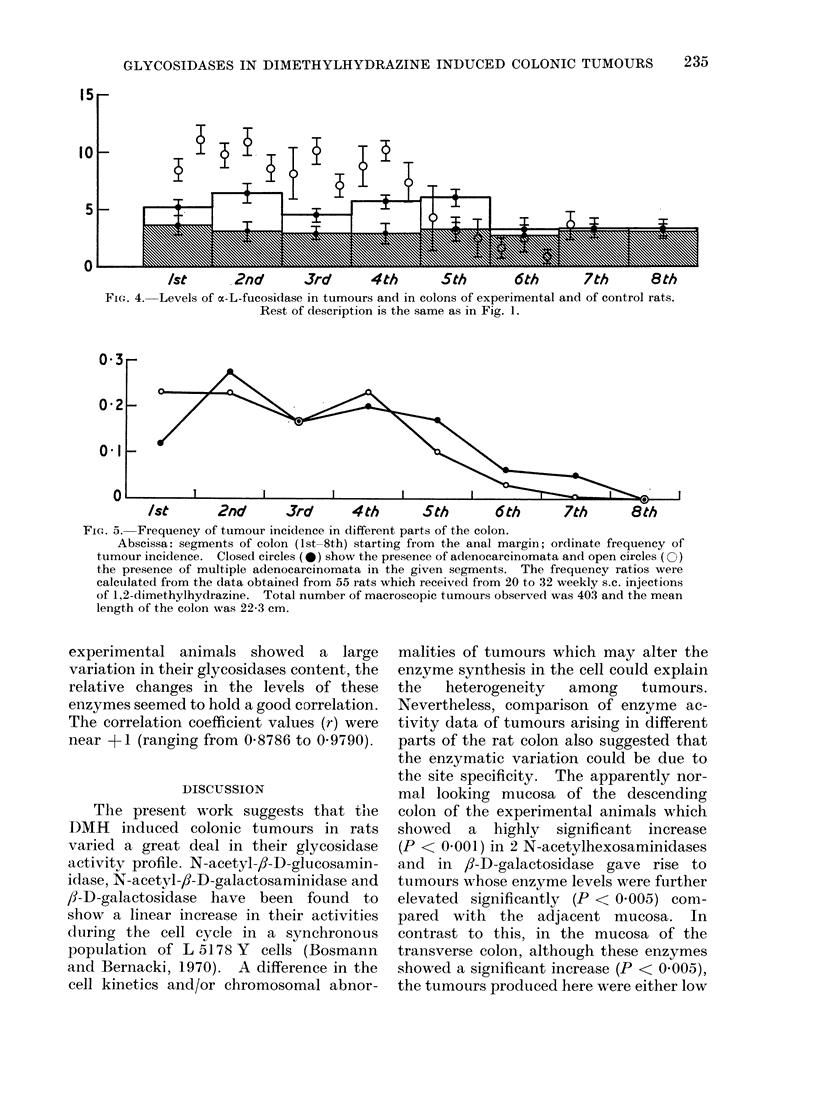

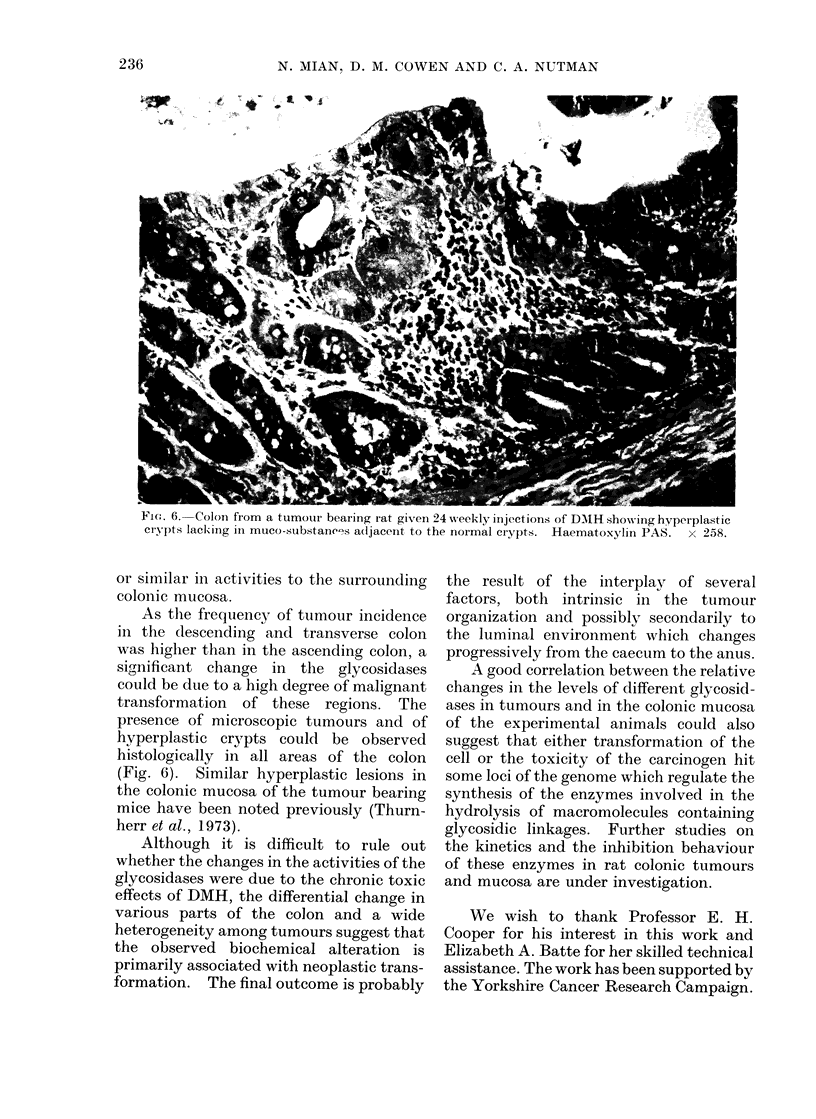

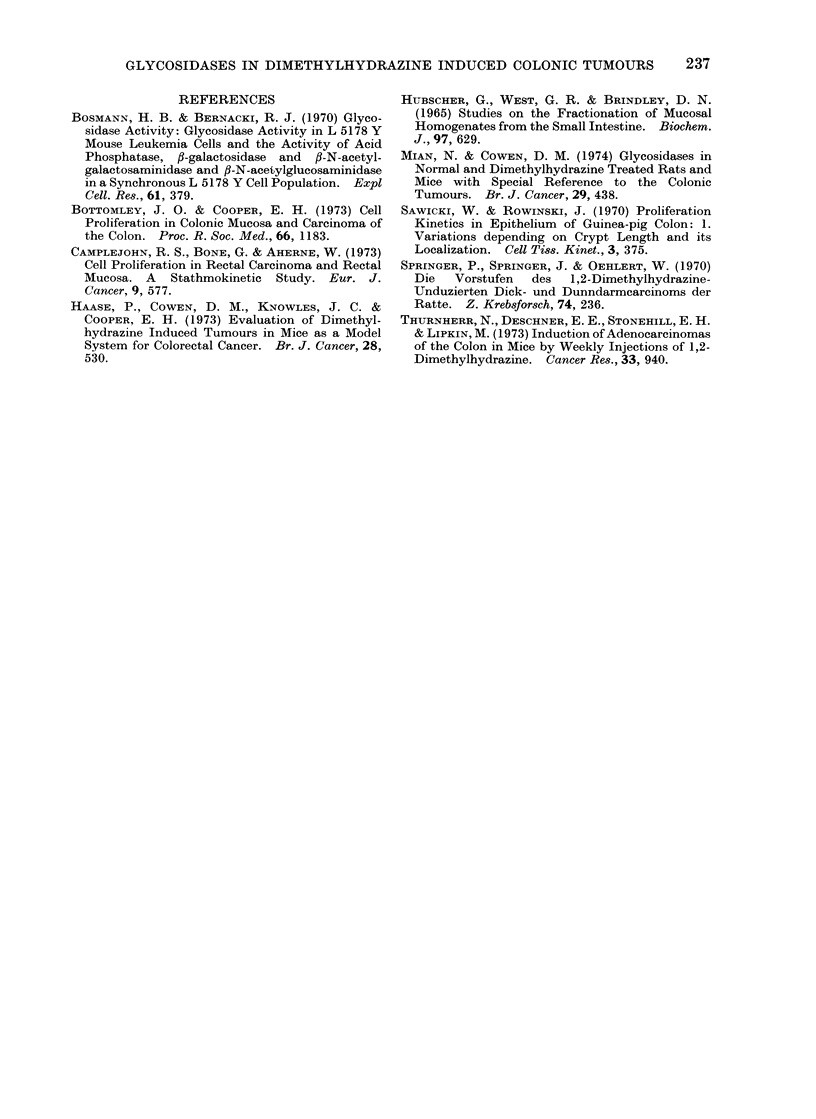


## References

[OCR_00520] Bosmann H. B., Bernacki R. J. (1970). Glycosidase activity. Glycosidase acitivity in L5178Y mouse leukemic cells and the activity of acid phosphatase, beta-galactosidase and beta-N-acetylgalactosaminidase and beta-N-acetylglucosaminidase in a synchronous L5178Y cell population.. Exp Cell Res.

[OCR_00529] Bottomley J. P., Cooper E. H. (1973). Cell proliferation in colonic mucosa and carcinoma of the colon.. Proc R Soc Med.

[OCR_00534] Camplejohn R. S., Bone G., Aherne W. (1973). Cell proliferation in rectal carcinoma and rectal mucosa. A stathmokinetic study.. Eur J Cancer.

[OCR_00540] Haase P., Cowen D. M., Knowles J. C., Cooper E. H. (1973). Evaluation of dimethylhydrazine induced tumours in mice as a model system for colorectal cancer.. Br J Cancer.

[OCR_00547] Hübscher G., West G. R., Brindley D. N. (1965). Studies on the fractionation of mucosal homogenates from the small intestine.. Biochem J.

[OCR_00553] Mian N., Cowen D. M. (1974). Glycosidases in normal and dimethylhydrazine-treated rats and mice with special reference to the colonic tumours.. Br J Cancer.

[OCR_00559] Sawicki W., Rowiński J. (1970). Proliferation kinetics in epithelium of guinea-pig colon. I. Variations depending on crypt length and its localization.. Cell Tissue Kinet.

[OCR_00565] Springer P., Springer J., Oehlert W. (1970). Die Vorstufen des 1,2-Dimethylhydrazin-induzierten Dick- und Dünndarmcarcinoms der Ratte.. Z Krebsforsch.

[OCR_00571] Thurnherr N., Deschner E. E., Stonehill E. H., Lipkin M. (1973). Induction of adenocarcinomas of the colon in mice by weekly injections of 1,2-dimethylhydrazine.. Cancer Res.

